# Allelic Variation of *BnaC*.*TT2*.*a* and Its Association with Seed Coat Color and Fatty Acids in Rapeseed (*Brassica napus* L.)

**DOI:** 10.1371/journal.pone.0146661

**Published:** 2016-01-11

**Authors:** Longhua Zhou, Yuanlong Li, Nazim Hussain, Zhilan Li, Dezhi Wu, Lixi Jiang

**Affiliations:** College of Agriculture and Biotechnology, Zhejiang University, Hangzhou, 310058, People’s Republic of China; Huazhong university of Science and Technology, CHINA

## Abstract

Efficient molecular markers for the selection of rapeseed genetic materials with high seed oil content and ideal fatty acid (FA) composition are preferred by rapeseed breeders. Recently, we reported the molecular mechanism of *TRANSPARENT TESTA 2* (*TT2*) in inhibiting seed FA biosynthesis in *Arabidopsis*. However, evidence showing the association of rapeseed *TT2* homologs and seed FA production are still insufficient. In this study, we collected 83 rapeseed (*Brassica napus* L.) landraces from different geographical backgrounds to conduct association mapping of *BnaC*.*TT2*.*a* in relation to seed coat color and FA biosynthesis. Population background was corrected by 84 pairs of SSR markers that were uniformly distributed among the linkage groups of the Tapidor-Ningyou-7 DH population. A single copy of *BnaC*.*TT2*.*a* for single nucleotide polymorphism (SNP) assay was cloned by a pair of previously reported specific primers. From the analysis of *BnaC*.*TT2*.*a* allelic variations using GLM+Q model, four SNPs on intron 1 of *BnaC*.*TT2*.*a* that were associated with seed FA were discovered. Moreover, an *InDel* at position 738 on exon 3 of *BnaC*.*TT2*.*a* indicated a change of protein function that was significantly associated with seed coat color, linoleic acid (C18:2), and total FA content. These findings revealed the role of *BnaC*.*TT2*.*a* in regulating the seed color formation and seed FA biosynthesis in rapeseed, thereby suggesting effective molecular markers for rapeseed breeding.

## Introduction

Rapeseed (*Brassica napus* L.) is a globally important oilseed crop ranked third after soybean and oil palm. Rapeseed breeders deal with both quantitative and qualitative traits for genetic improvement. However, most of the traits relating to seed yield and seed quality are quantitative. Numerous research efforts have been invested into obtaining favorable agronomic traits, such as high total seed fatty acids (FA) and high proportion of unsaturated 18C FAs (i.e., oleic and linoleic acids), simultaneously. Quality degrading traits, such as seed erucic acid, thioglycoside, color substances, and crude fiber need to be reduced [1].

At present, the majority of commercially available *B*. *napus* seeds vary in color from brown to dark brown or black [1–3]. However, yellow-seeded varieties have become interesting to researchers because of their decreased thickness, lignin content, and defective proanthocyanidin (PA) biosynthesis. The superior characteristics of yellow seeds result in low pigment deposition in the seed coats, high proportion of seed oil, and synthesis of meal protein [4, 5]. At present, *Arabidopsis* mutants with several defective flavonoid pathway genes have been identified and correlated with yellow to pale brown seed coat color. These mutants are referred to as *transparent testa* (*tt*), which includes *tt1*, *tt2*, *tt4*, *tt5*, *tt6*, *tt7*, *tt8*, *tt10*, and *tt12*. These mutants contain higher seed oil than the wild type [6]. In the studies that aimed to elucidate the metabolic pathway of flavonoids in *A*. *thaliana*, the complex of *TT2*, *TT8*, and *TTG1* could regulate the expressions of *TT3 (DFR)*, *TT18 (DLOX)*, *TT12 (MATE)*, and *ANR*, which are the key factors affecting the seed coat color [7–10]. More recently, it was found that *TT2* and *TT8* could target *FUS3*, *LEC1*, *LEC2*, and *CDS2* to regulate seed development and/or oil biosynthesis [6, 11]. In the past, key studies on the *transparent testa* gene family in *Brassica* species include the cloning of *BnTT2* and *BnTTG1* [12–15] in *B*. *napus* and the alteration in seed color through insertion of the *BrTT8* transcription factor in *B*. *rapa* [16]. These studies provide a basis for the association between these genes and seed coat color/FA in rapeseed. The stability of yellow-seeded phenotypes in diploid *Brassica* species, such as *B*. *rapa* and *B*. *oleracea*, is not rare. However, yellow-seeded types in *B*. *napus* do not occur naturally [17]; these types are developed from interspecific crosses with related diploid progenitor species, such as *B*. *rapa*, *B*. *oleracea*, *B*. *juncea*, and *B*. *carinata* [17, 18]. Hence, to get insight into the molecular mechanism regulating seed color is important for the successful breeding of stable yellow-seeded cultivars of *B*. *napus*.

In *Brassica*, mapping of quantitative trait loci (QTL) in segregating populations is well documented and has been used to analyze and identify QTL for seed coat color [19–21] and other important traits [22–25]. Although QTL mapping helps identify and estimate the contributive effects of loci on a quantitative trait, it still has several inherent disadvantages. QTL mapping still requires a specific mapping population and can only calculate the allelic diversity for the cross between two parents with different alleles controlling a trait. Therefore, QTLs cannot be identified for the parents both have same allele [26]. Moreover, the limitation in number of recombination events in a segregating population could impose restriction to the resolution of QTL mapping. This limitation leads to variation in confidence intervals for the QTL positions, ranging from a few cM up to several tens of cM [27, 28].

Linkage Disequilibrium (LD) mapping, which is also called association mapping or linkage mapping, is a potentially alternative approach to QTL mapping. LD mapping probes the associations between marker(s) and trait(s) by studying the degree of LD between markers and functional polymorphisms across diverse germplasm [29, 30]. The main advantages of LD mapping over the QTL mapping technique include improved mapping resolution, reduced research time, and greater allele number [31]. Hence, the use of modern genetic technologies and methods could help researchers to use natural diversity in large gene pools of plant species and locate valuable genes in the genome [29, 30, 32]. LD measures the extent of non-random association between alleles at different loci or regions of the genome that are inherited together at a frequency higher than expected based on recombination [30]. Single nucleotide polymorphism(SNP), with moderate or high LD frequency, may extend over the length of genes or gene clusters that could possibly determine the SNP haplotypes. Identification of the trait(s) associated with SNPs using LD decay in *Arabidopsis* could provide a powerful data set for evolutionary and genetic association studies [33]. Moreover, LD assists breeders to screen large germplasm with potentially powerful and fast-tracked breeding program methods for crop improvement [34].

*TT2* regulates PA biosynthesis, and/or tannin accumulation in plant seeds. Moreover, *TT2* could play a vital role in seed development and regulate the expression of numerous genes involved in the FA biosynthesis pathway by targeting *FUSCA3* in *Arabidopsis* [6]. In association analyses between markers and traits in canola type rapeseed, LD decay resulted in significantly higher resolution than QTL analyses in segregating populations, as shown by interval mapping [35]. So far, in *B*. *napus* only a few studies have been conducted regarding the association of phenotypes and candidate genotype governing genes. For instance, it was found there are 26 and 12 allelic polymorphisms at two loci, namely *BnaX*.*VTE3*.*a*, *BnaA*.*PDS1*.*c*, respectively, responsible for changes of seed tocopherol content [36]. Moreover, association analysis of *BnaA*.*FRI*.*a* in 248 *B*. *napus* accessions revealed significant correlation between six SNPs sites and variation of flowering time [37].

Given the functional attributes of *TT2* in flavonoids and seed oil synthesis in *Arabidopsis*, we evaluated the allelic variation of *BnaC*.*TT2*.*a* in a diverse collection of rapeseed (*B*. *napus*) landraces, and associated its genetic polymorphism with seed color and FA content using association mapping technique.

## Materials and Methods

### Ethics statement and plant materials

Ethics statement is “N/A” as the study was conducted on plants. The field used for the experiment was Huajiachi Agricultural Farm which is the property of Zhejiang University, Hangzhou, P.R. China and professors of the university are allowed to use the land for their research work. Thus, no specific permissions were required. Moreover, a collection of 83 rapeseed genotypes (*B*. *napus* L.) with various genetic backgrounds were obtained mainly from the Department of Plant Breeding and Genetics, The University of Agriculture, Peshawar, Pakistan (49, the majority of which are local varieties), 28 Chinese local varieties were provided by Prof. Wu Xiaoming of Institute of Oil Crops, Chinese Academy of Agricultural Sciences. The genotypes with genetic backgrounds from Germany, Canada, and France were obtained from the genebank of the IPK Gatersleben, Germany (Table 5). Furthermore, we confirm that the field studies did not involve endangered or protected species. A field experiment in a randomized complete block design with three replications was carried in the growing season 2010–2011. Initially, an inflorescence of each genotype was randomly tagged and covered with bags for selfing. The seeds from the upper raceme of the main branch or from the uppermost side branch were collected for the analysis of FAs.

### Seed coat color assessment

The seed coat color was interpreted according to the Color Index for Red Grapes (CIRG) color criteria, following the method of Shen et al., [38] and by using Minisan XE PLUS (USA Hunter Associate Laboratory, Inc.). These color traits were expressed in terms of tristimulus indices, namely, L*, a*, and b*. L* indicates lightness (100 = white and 0 = black), a* shows redness-greenness (positive = red), and b* presents yellowness-blueness (positive = yellow). Moreover, the chroma (C) value shows color intensity or saturation, and is calculated as C = (a^*2^ + b^*2^)^1/2^, whereas hue angle is calculated as H^o^ = tan^-1^(b*/a*). Seed coat color data was summarized using MS-Excel Windows software and the CIRG index presented by the Quantile-quantile (QQ) plot package in the R program.

### Fatty acid analysis

FA was analyzed following the methods described by Zou et al., [39] and Zhu et al., [40] with slight modifications. Briefly, 50 mg of the milled seed meal was homogenized with 2 mL of solution containing chloroform/isopropanol (2:1, v/v) in a 12 mL screw-top glass tube. Samples were kept in the dark for 2 h at room temperature and vigorous vortexing was done for 30 s per 30 min. Samples were then centrifuged at 2500 rpm for 5 min. The 400 μL supernatant was collected in a new tube and 2 mL of 1% MeOH/H_2_SO_4_ (v/v) was added to each tube. The tubes were warmed for 1 h in a water bath at 80°C. Furthermore, 2 mL of 0.9% NaCl was added to each sample after cooling the tubes at room temperature. Hexane was added thrice (1 mL each time) and samples were vortexed for the extraction of FA, followed by centrifugation at 2500 rpm for 2 min. Supernatant of about 700 μL was collected in GC vials, from which 2 μL were auto-injected into the gas chromatograph machine (SHIMADZU, Kyoto, Japan, GC-2014). The chromatograph machine was equipped with a flame ionization detector (FID) and a column (Supelco was-10, Schnelldorf, Germany) with length × inner diameter × liquid membrane thickness of 30 m × 0.25 mm × 0.5 μm, respectively. Temperature programming was configured as follows: an initial column temperature of 160°C for 1 min, which was then raised to 240°C at the rate of 4°C per min, and finally held for 16 min to end the analysis time set per sample. The peaks of FA species were identified according to their respective retention times. Moreover, the concentration of the individual peaks was normalized and quantified against methyl heptadecanoate, which was used as an internal standard and/or control. Total FA content was measured as the sum of all 11 FA species. Data was summarized using MS-Excel software and seed oil content presented by the QQ plot package in the R program.

### Population Structure

The population structure of 83 *B*. *napus* genotypes was shaped from 84 pairs of SSR markers, which were distributed uniformly in *B*. *napus* Tapidor-Ningyou-7 DH population. At least four pairs of markers were located at each linkage group. *B*. *napus* is an allopolyploid with a complex genome. Thus, one pair of SSR markers could result in multiple loci bands of a gene and eventually lead to non-targeted genotyping. Moreover, the reading of the bands obtained from SSR markers was based on their overall shape. The Q value of the population was obtained using the software STRUCTURE 2.3.4 (http://pritch.bsd.unchicago.edu/structure.html) [41]. Initially, we assessed the K values from 1 to 10, five times for each value, with length of burn-in period at 10,000 and Markov chain Monte Carlo at 100,000. The most appropriate groups were divided based on the two consecutive change rates of the *Ln p(D)* value. The K index corresponding to the peak Δk value was considered the number of taxa divisions [42].

### Genotypic analysis

DNA of all the 83 genotypes was extracted according to the CTAB method [43], and the concentration was set to 50 ng/μL. The sequence of candidate gene *BnaC*.*TT2*.*a* was obtained from the National Center for Biotechnology Information (NCBI) website. This gene was selected for association mapping based on its differential expression between yellow-seeded and black-seed rapeseed phenotypes, as well as its high similarity with Brassica *TT2* genes in the sequence database [15]. Gene-specific primer pairs were selected to amplify the single-gene product using PrimeSTAR HS DNA Polymerase (Takara Biotechnology [Dalian] Co., Ltd.). The amplification product was then purified by using 1% agarose gel electrophoresis. Amplification products of all 83 samples were subjected to Sanger sequencing (Shanghai Sunny Biotechnology Co., Ltd.). Sequences of all the 83 DNA samples were compared by the sequence alignment using CLASTALW2 software. The sequence alignment results were then subjected to DNA polymorphism analysis at the locus *BnaC*.*TT2*.*a* among the 83 genotypes using TASSEL software (Version 3.0). To identify significant SNPs/indel, threshold value of the minor allele frequencies (MAF) was set to 5%. Moreover, the genotype based haplotype groups evaluation was conducted according to the diversity of SNP observed for each genotype.

### Association analysis

Using the TASSEL 3.0 software, DNA polymorphism was further analyzed to identify the association between color traits, including CIRG, L*, a*, b*, C, and H°, seed FA composition, and total FA [44]. Analysis for association mapping was performed using the GLM + Q model and the population structure were estimated using the Q-matrix function of the STRUCTURE software. Moreover, the level of significance was set to (Bonferroni correction) P≤0.05 for the correlation analysis of the characteristics. Significance of the linkage between the polymorphismic sites and the traits were interpreted and characterized based on *P* and *R*^*2*^ values, respectively.

## Results

### Distribution of seed coat color and seed fatty acids

**Fig 1:**
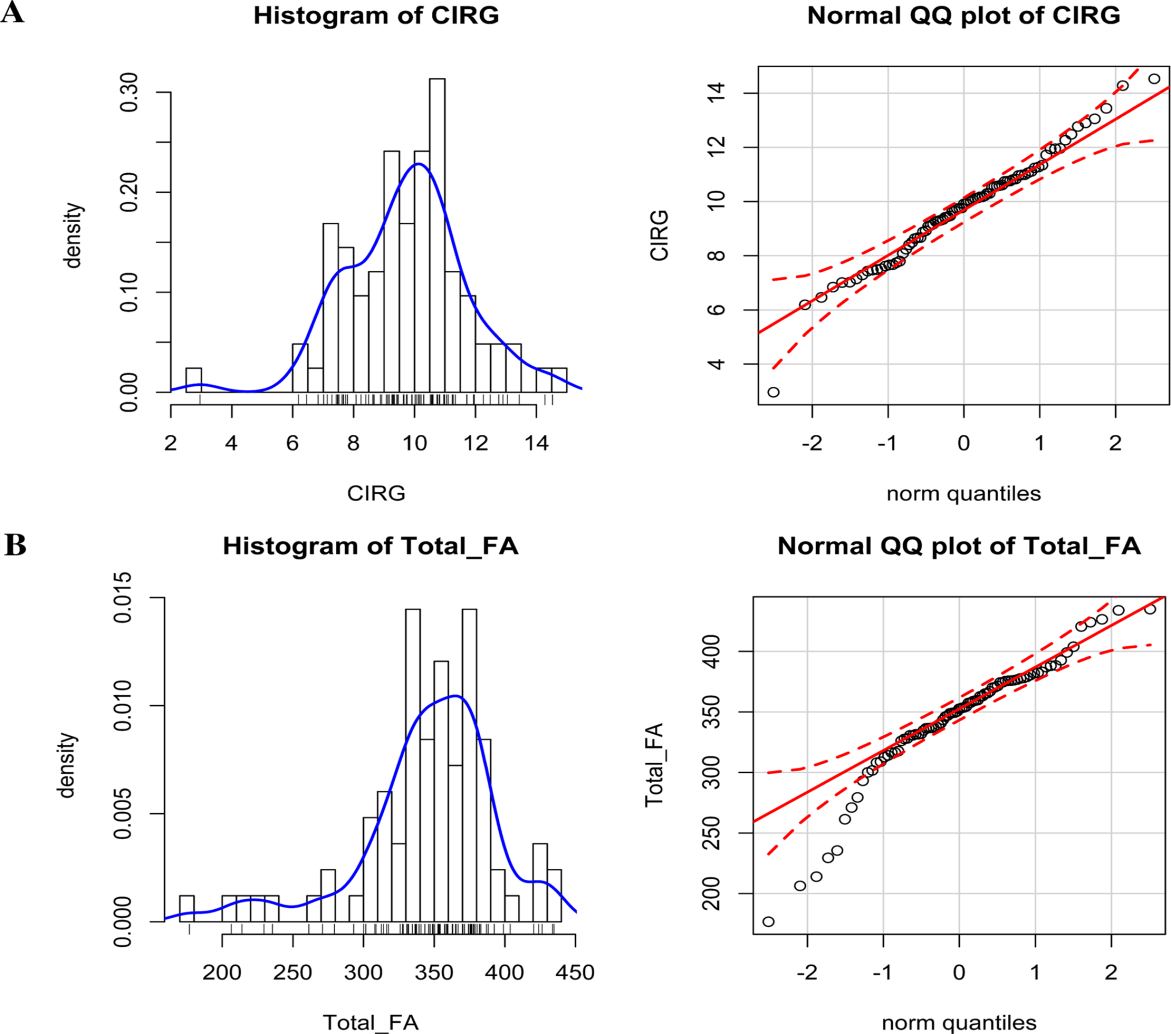
Seed coat color and total seed FA content of 83 genotypes of *B*. *napus* L. (A) Shows the normal distribution and QQ plot configuration of the CIRG, whereas, (B) shows the normal distribution and QQ plot configuration of seed total FA, respectively.

**Fig 2:**
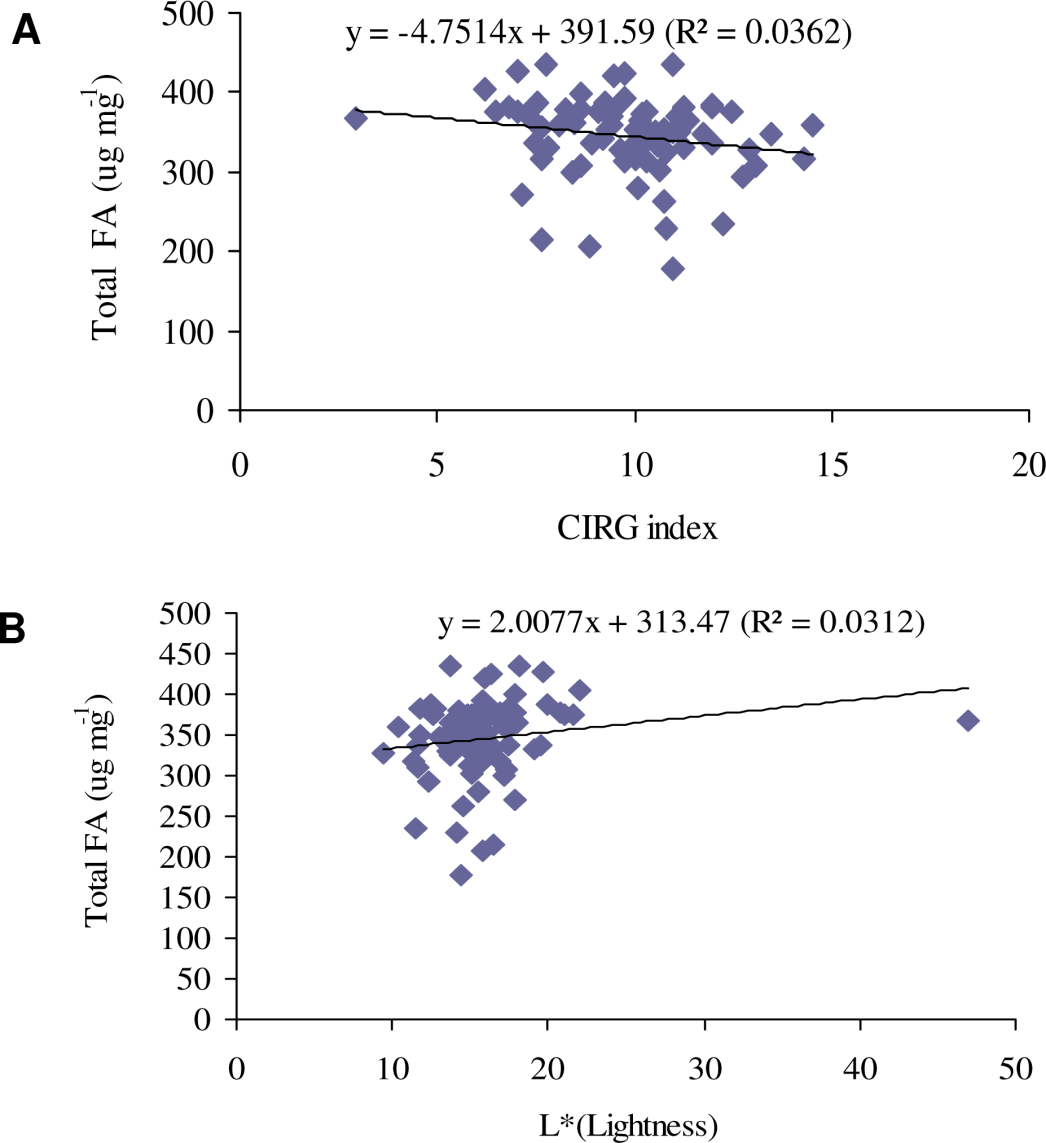
Correlation analysis between the CIRG or L and the total fatty acids (values for total FA are represented as μg mg^-1^).

Phenotypic diversity in terms of seed color and FA contents in all 83 genotypes was observed and measured. QQ plot analysis revealed normal data distribution from the analysis of seed coat color traits (Fig 1A) and seed FA content (Fig 1B). We investigated the relationship between seed coat color and seed FA. Color measurements, expressed as tristimulus parameters, revealed that CIRG had a significantly negative correlation with FA composition (C18:0, C18:1, and C18:2) and total seed FA (Table 1, Fig 2A). Moreover, the lightness (L*) of the seed coat had a significantly positive correlation with seed FA composition (C18:0 and C18:2) and total seed FA (Table 1, Fig 2B). The other significantly positive correlations found were a* with C18:2, b* with C18:1 and C18:2, and C with C18:0, C18:1, and C18:2. A non-significant correlation was observed among H°, total FA, and FA composition (Table 1).

**Table 1:** Correlations among tristimulus parameters for seed coat color and the FA contents and compositions in *B*. *napus*L.

Kendall’s tau b	CIRG	L*	a*	b*	C	H^o^
**C18:0**	-0.17*	0.15*	.	.	0.17*	.
**C18:1**	-0.15*	.	.	0.16*	0.18*	.
**C18:2**	-0.16*	0.15*	0.17*	0.18*	0.18*	.
**C18:3**	.	.	.	.	.	.
**C22:1**	.	.	.	.	.	.
**Total FA**	-0.17*	0.17*	.	.	.	.

*, significant at P<0.05;^.^, non-significant; CIRG, Color Index for Red Grapes; Letters L*, a*, b* and C indicate the tristimulus parameters for the seed coat color. L*, lightness; a*, redness-greenness (positive = red); b*, yellowness-blueness (positive = yellow); H^o^, hue angle.

### Population structure (Q) and phenotype of the subpopulation

**Fig 3:**
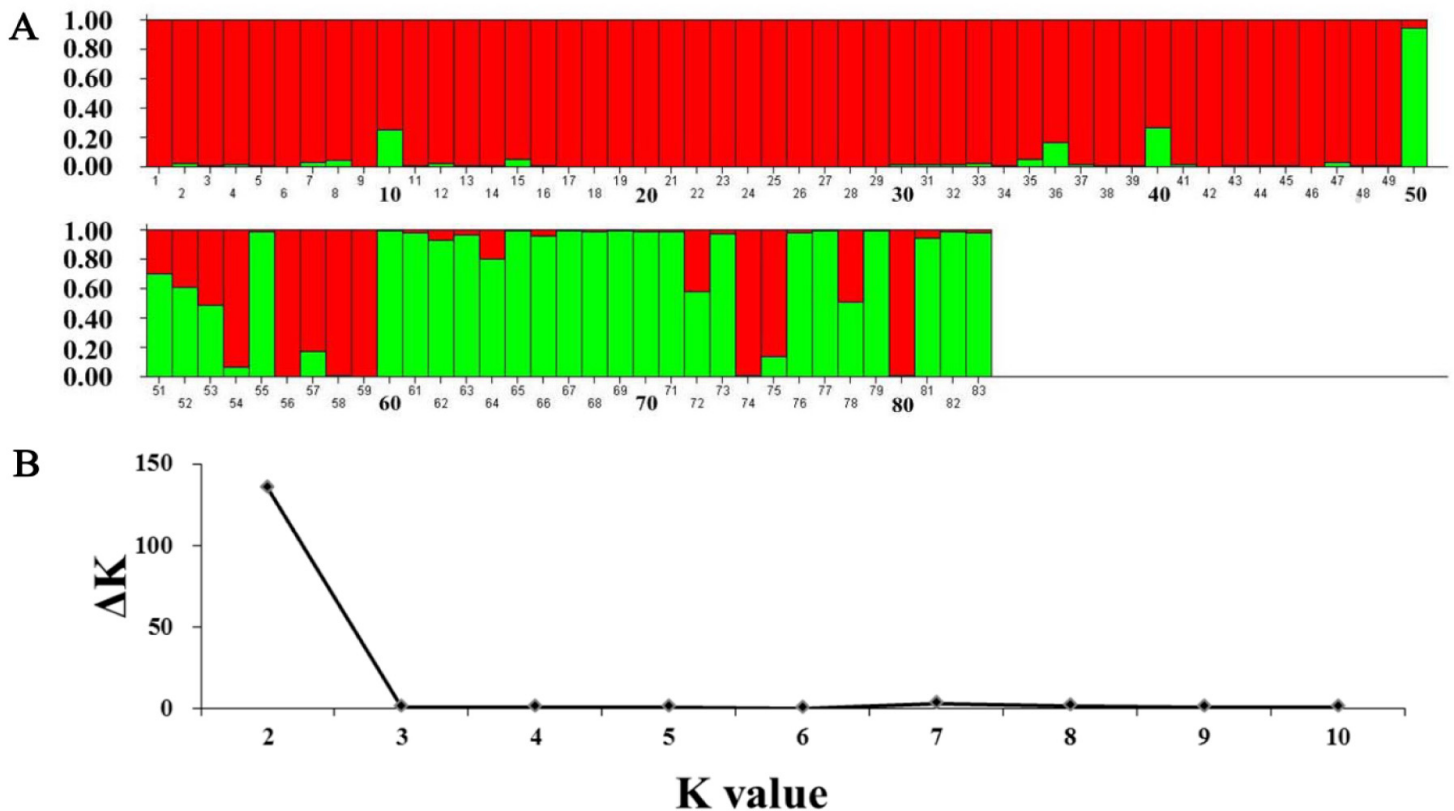
(A) Evaluation of population structure of 83 *B*. *napus* accessions based on 84 SSR markers. (B) The related estimation of *K* subpopulation. The accessions are represented by bars which could be divided into two categories based on their colors. X-axis indicates the number of accessions and Y-axis shows the percentage of group membership.

Population (POP) structure of 83 *B*. *napus* accessions was analyzed using 84 SSR markers. These SSR markers were distributed across the genetic map of *B*. *napus* and divided the population into two subpopulations (POP1 and POP2) based on their genetic diversity, as shown in the bar colors (Fig 3A). Red bars showing POP1 included 58 genotypes, whereas POP2 included 25. Moreover, the number of subpopulations suitable for association analysis was estimated by applying the Δk criterion. The distribution of Δk relative coefficients revealed significant variation in the likelihood of 83 *B*. *napus* accessions, ranging from 1 to 10 (Fig 3B). The most evident and highest likelihood for a subpopulation was observed with k = 2 and *Ln p(D)* = -10,523.7, with a variance of 897.26, using the STRUCTURE software (Fig 3B, Table 2). Furthermore, data on tristimulus parameters (color traits), and FA analysis revealed that POP2 had greater mean lightness (L*), redness (a*), and chroma (C) values than POP1. On the other hand, POP1 had higher yellowness (b*) and H^o^ values than POP2. Overall, POP1 had higher seed oil content than POP2, but this change was statistically non-significant. POP2 had higher C18 FAs, such as C18:0, C18:1, C18:2, and C18:3; and lower long-chained but mostly undesirable FA content, including C20:0, C20:1, C22:0, C22:1, and C24:0 than POP1 (Table 3).

**Table 2:** Calibrated background in 83 genotypes of *B*. *napus* using 84 pairs of SSR markers.

K	*Ln p(D)*	Variance
1	-10807	277.14
2	-10523.7	897.26
3	-19155.8	18216.28
4	-17563.4	15692.08
5	-28450.3	37120.38
6	-25902.1	32443.98
7	-22243.5	25538.26
8	-73196.6	127283.8
9	-38271.2	57070.98
10	-64016.7	109031

*Ln p(D)*, a natural logarithm of the probability of the data that determines the value K, whereas, K is the index showing the number of subpopulations suitable for association analysis.

**Table 3:** Comparison of the seed coat color and fatty acid between two subpopulations of *B*. *napus*.

		CIRG	L*	a*	b*	C	H^o^	C18:0	C18:1	C18:2	C18:3	C20:0	C20:1	C22:0	C22:1	C24:0	TotalFA
**POP1**	Mean	9.87	15.78	2.75	1.77	3.53	0.49	4.87	106.8	52.33	26.6	2.64	41.36	1.73	96.99	0.86	346.65
	CV(%)	19.66	30.36	46.88	112.1	58.91	60.45	21.29	39.77	16.44	17.68	22.3	38.37	40.13	49.7	70.77	13.16
**POP2**	Mean	9.43	16.1	3.14	1.73	3.74	0.47	5.23	122.1	57.27	30.87	2.22	22.05	1.72	87.5	0.78	342.31
	CV(%)	20.56	16.79	49.26	77.99	48.04	57.17	51.01	75.8	33.98	14.48	22.51	66.78	41.61	80.94	40.52	16.1

Letters L*, a*, b* and C indicate the tristimulus parameters for the seed coat color. L*, lightness; a*, redness; b*, yellowness; C, chroma value indicates color intensity or saturation; H^o^, hue angle; POP1, population 1; POP2, population 2.

### Nucleotide polymorphism and haplotype diversity

To determine the nucleotide(s) variation of *BnaC*.*TT2*.*a* in a panel of the 83 genotype, a single primer pair, namely, *BnaC*.*TT2*-L 5ʹ-TTGATAGCTGGGAGGCTTCCAGG-3ʹ and *BnaC*.*TT2*-R 5ʹ-CCAAACCATCAAAGCCCATTAA-3ʹ [15], was used to amplify the coding sequence of the *BnaC*.*TT2*.*a* gene. SNPs were detected using the alignment of sequencing results, and the entire population was categorized into various haplotypes. Sequence of *BnaC*.*TT2*.*a* gene with accession number “DQ778645.1” was used as a reference to categorize the population into haplotypes. We found seven haplotype groups (H) based on the SNPs detected across the entire population of *B*. *napus* (Table 4). Analyses revealed that among the population of 83 *B*. *napus* genotypes, haplotype group H3 presented the most (34 genotypes) members of the population, followed by H0, H4, H2, H1, H5, and H6 with 29, 8, 6, 3, 2, and 1 numbers of genotypes, respectively (Table 5). SNPs detected were both monomorphic as in the case of H1 and H2, as well as polymorphic as shown by H0, H3, H4, H5, and H6.

**Table 4:** Single nucleotide polymorphism (SNP) detected among 83 genotypes of *B*. *napus* on *BnaC*.*TT2*.*a* gene locus.

	*BnaC.TT2.a* (5' to 3')						GeneBank ID
	166	188	222	226	738	790	
**H0**	A	A	A	C	T	T	DQ778645.1
**H1**	·	·	·	·	·	G	
**H2**	T	·	·	·	·	·	
**H3**	T	G	C	T	·	·	
**H4**	T	G	C	T	·	G	
**H5**	T	G	C	T	*Indel*	·	
**H6**	T	G	C	T	*Indel*	G	

H, Haplotype; *Indel*, insertion and/or deletion

**Table 5:** Distribution of 83 genotypes of *B*. *napus* into different haplotype groups on the basis of population structure analysis at *BnaC*.*TT2*.*a* gene locus.

A.N.	Origin	Haplotype	A.N.	Origin	Haplotype	A.N.	Origin	Haplotype	A.N.	Origin	Haplotype
1	Rajanpur, PK	H3(POP1)	22	Dara, PK	H3(POP1)	43	Risalpur, PK	H0(POP1)	64	IOC 2w019, Hunang, CN	H0(POP2)
2	Pakpattan, PK	H4(POP1)	23	Swabi, PK	H0(POP1)	44	Chamkani, PK	H0(POP1)	65	Gan-lan-xing 077, Hubei, CN	H6(POP2)
3	Swabi, PK	H0(POP1)	24	Rawalpindi, PK	H3(POP1)	45	Tarnol, PK	H3(POP1)	66	Luo-jing-xuan-xi, Shanghai, CN	H3(POP2)
4	Nowshera, PK	H4(POP1)	25	Tank, PK	H2(POP1)	46	Wazir abad, PK	H3(POP1)	67	IOC 79–1, Shanghai, CN	H5(POP2)
5	Mardan, PK	H1(POP1)	26	Islamsbad, PK	H0(POP1)	47	Shabqadar, PK	H0(POP1)	68	R-line 4190, Shanghai, CN	H3(POP2)
6	Bannu, PK	H3(POP1)	27	Haripur, PK	H3(POP1)	48	Khairabad, PK	H3(POP1)	69	IOC 78251, Zhejiang, CN	H3(POP2)
7	Attock, PK	H0(POP1)	28	Nowshera, PK	H0(POP1)	49	Kurram Agency, PK	H3(POP1)	70	IOC 135, Anhui, CN	H3(POP2)
8	Lasbela, PK	H4(POP1)	29	Batgram, PK	H3(POP1)	50	Zhe-shuang 72, Zhejiang, CN	H0(POP2)	71	IOC 135, Shanghai, CN	H3(POP2)
9	Laki, PK	H0(POP1)	30	Charat, PK	H0(POP1)	51	Zhe-da 619, Zhejiang, CN	H0(POP2)	72	IOC cuan-205, Sichuan, CN	H0(POP2)
10	Taxila, PK	H3(POP1)	31	Mianwali, PK	H0(POP1)	52	Zhe-shuang 758, Zhejiang, CN	H2(POP2)	73	IOC 3145–20, Hunang, CN	H0(POP2)
11	Hassan Abdal, PK	H3(POP1)	32	Bannu, PK	H4(POP1)	53	Hu-you-qing, Shanghai, CN	H0(POP1)	74	Mei-jian, Xizang, CN	H0(POP1)
12	Kohat, PK	H2(POP1)	33	Nowshera, PK	H0(POP1)	54	Falcon, DE	H3(POP1)	75	Fu-you 4, Heilongjiang, CN	H0(POP1)
13	Kusur, PK	H0(POP1)	34	Laki, PK	H3(POP1)	55	Gao-you 605, Zhejiang, CN	H3(POP2)	76	Fu-you 2, Fujian, CN	H1(POP2)
14	Dado kandao, PK	H3(POP1)	35	Charsadda, PK	H2(POP1)	56	ap-NPZ, DE	H3(POP1)	77	IOC 7515, Jiangxi, CN	H3(POP2)
15	Mardan, PK	H3(POP1)	36	Charat, PK	H3(POP1)	57	Ap-tengbe, DE	H4(POP1)	78	Xiang 85–16, Hunang, CN	H4(POP2)
16	Abba khel, PK	H1(POP1)	37	Narowal, PK	H4(POP1)	58	Ap-rakow, CA	H0(POP1)	79	75–1, Jiangxi, CN	H3(POP2)
17	Peshawar, PK	H2(POP1)	38	Okara, PK	H2(POP1)	59	Ap-renavd, FR	H3(POP1)	80	Westa, CA	H0(POP1)
18	Islamsbad, PK	H0(POP1)	39	Dara, PK	H3(POP1)	60	IOC 84–24016, Shanghai, CN	H4(POP2)	81	Zhong-shuang 6, Hubei, CN	H0(POP2)
19	TakhtNusrati, PK	H3(POP1)	40	Rawalpindi, PK	H0(POP1)	61	Xu-you 1, Jiangxi, CN	H5(POP2)	82	Zhong-shuang 9, Hubei, CN	H0(POP2)
20	Narowal, PK	H0(POP1)	41	Batgram, PK	H3(POP1)	62	IOC 106, Henang, CN	H0(POP2)	83	Xin-yang 7833, Henan, CN	H3(POP2)
21	Okara, PK	H3(POP1)	42	Swat, PK	H3(POP1)	63	IOC 5708A, Hunang, CN	H3(POP2)			

A.N, accession number

### Phenotypic differences among different haplotypes

**Fig 4:**
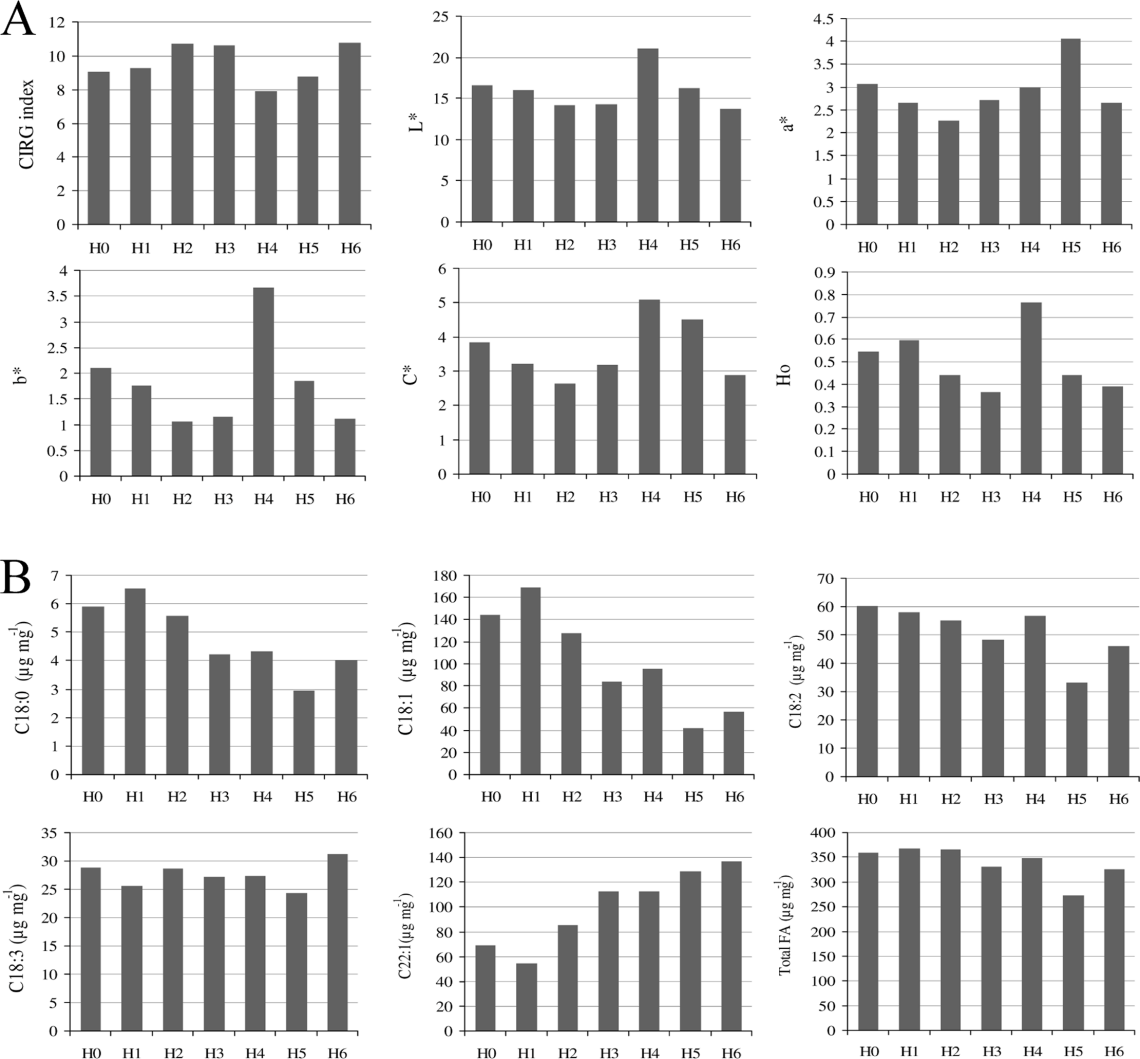
Comparison among different haplotypes of *B*. *napus* on the basis of; (A) tritimulus parameters of seed coat color and (B) seed fatty acids content.

Individual comparison among different haplotypes based on seed coat color and FA content and/or composition, revealed some interesting findings. For instance, the H4 haplotype has a relatively high L*, b*, C*, and H^0^ index values except for a*, suggesting the plausible functional attributes of *TT2* gene in changing the seed coat color (Fig 4A). Conversely, haplotypes H5 and H6 have FA composition with higher C22:1, but with lower C18 derivatives and total FA contents compared with other haplotypes. Thus, these genotypes have relatively low seed oil content. We found that in C22:1, which may negatively affect human health, H0 and H1 haplotypes have relatively low C22:1, but higher C18:0, C18:1, and C18:2 FA derivatives compared with other haplotypes. Thus, these are a candidate germplasm pool for oilseed breeding programs (Fig 4B).

### Linkage disequilibrium at the *BnaC*.*TT2*.*a* locus

**Fig 5:**
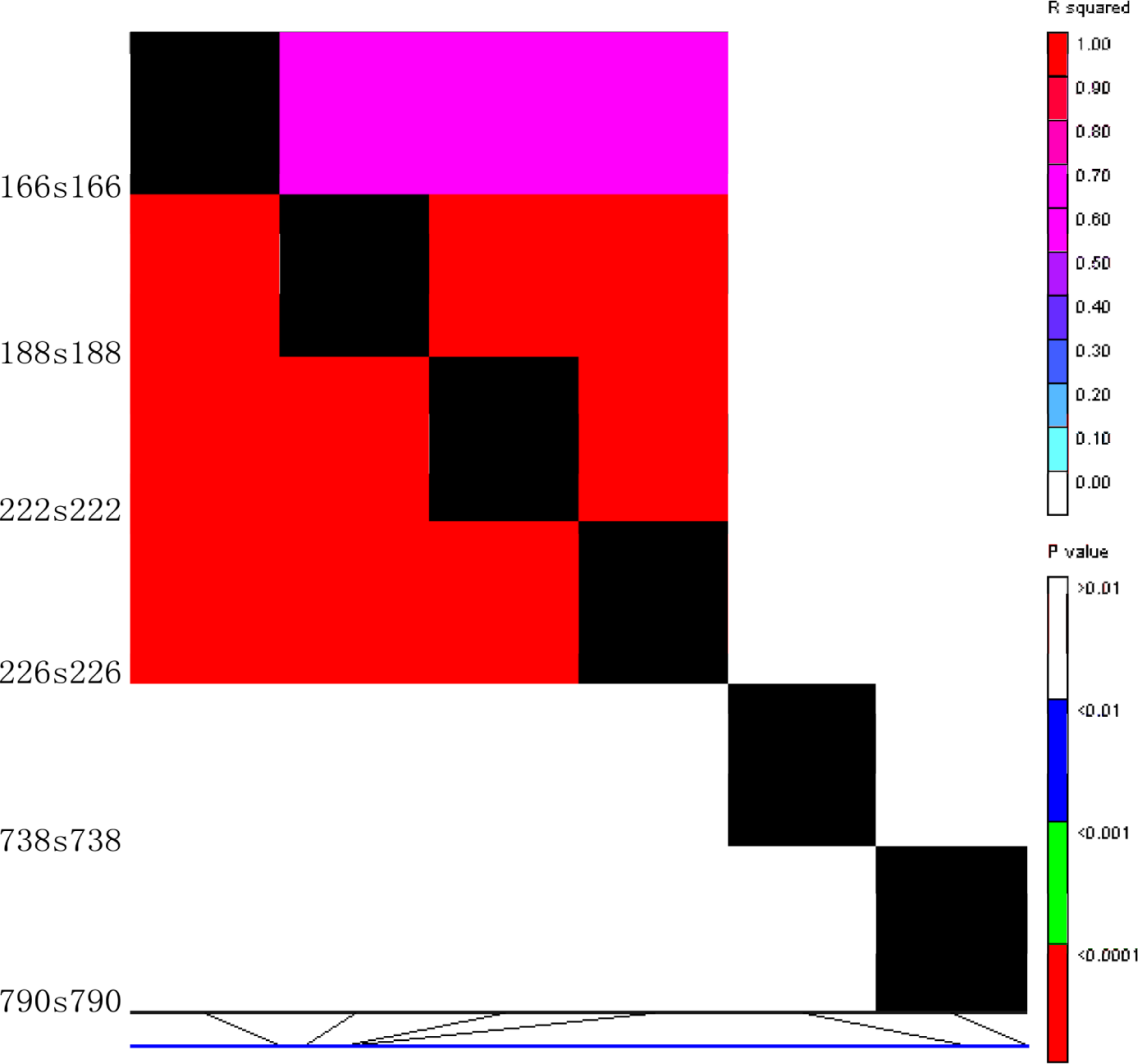
Linkage disequilibrium of 83 *B*. *napus* genotypes obtained on the basis seed color traits, seed fatty acids and single nucleotide polymorphism at *BnaC*.*TT2*.*a* gene locus.

**Fig 6:**
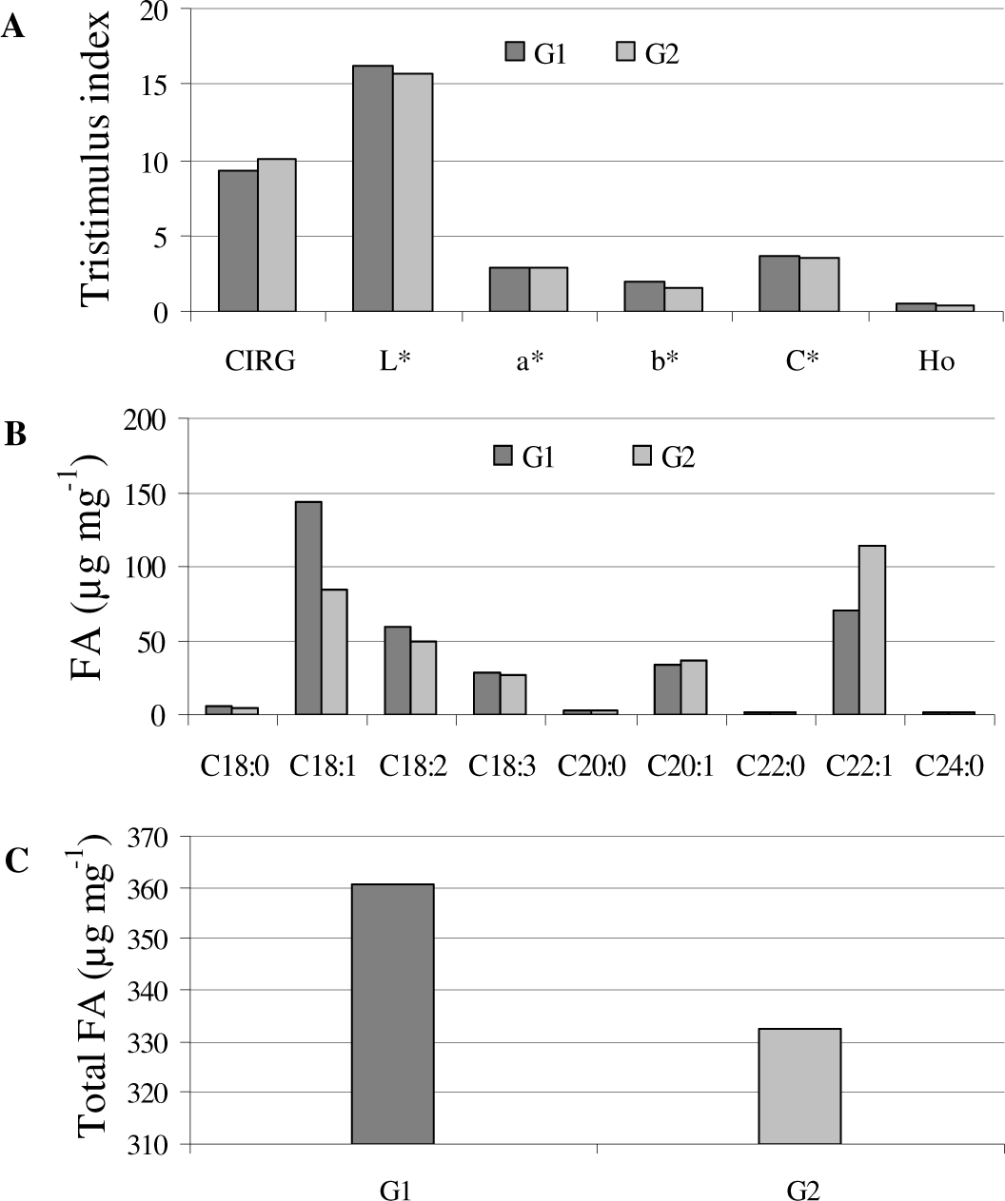
Comparison between two subgroups of hyplotypes based on significant association of SNPs to the seed coat color and/or FA traits. G1, represents H0, H1, and H2, whereas, G2 represents H3, H4, H5 and H6.

All the 83 *B*. *napus* accessions were genotyped at *BnaC*.*TT2*.*a* locus by PCR amplification from genomic DNA with single primer pair and subsequently subjected to sequencing. The primer covers the gene from the start codon to 890 bp. To analyze the degree of LD within *BnaC*.*TT2*.*a*, *R*^2^ values for each pair of polymorphism was determined using TASSEL software (v3.0) [44]. Only SNPs with frequencies ≥ 0.05 were considered for LD decay analysis. In total, five SNPs and one *Indel* were identified. Of these six polymorphic sites, four were located within introns and two within the coding sequence. P<0.001 was observed between SNP 166 and SNP 226 for the *BnaC*.*TT2*.*a* polymorphism (Fig 5), spanning four introns. Moreover, the population was structured into seven different haplotypes, which were further categorized based on the significant outcomes from LD analysis. From the four SNPs (gene position 166, 188, 222, and 226) that have significant phenotypic to genotypic trait associations depicted from the LD map (Fig 5), we grouped the haplotypes H0, H1, and H2 as G1. The haplotypes H3, H4, H5, and H6, having four common and significant linkage SNPs at these polymorphic sites, were grouped as G2. We found that genotypes of G2 have comparatively lower oil content (approx. 8.4%) than those of G1 (Fig 6A). Moreover, the oil quality of G2 was inferior to G1 in terms of higher components of C22:1 and lower components of C18:2 and C18:1 (Fig 6B). Non-significant variations were observed in the seed coat color of both G1 and G2 (Fig 6C).

### Association analysis between genotypic and phenotypic traits

Polymorphism observed in DNA samples were analyzed for association with seed color and FA traits using the GLM + Q model. Polymorphic sites with minor allele frequencies greater than 5% were used for the association analysis. In total, 28 significant associations (P<0.05) were observed for six polymorphic sites (Table 6) in *BnaC*.*TT2*.*a* locus. We found that C18:1, C18:2, C22:0, and C22:1 and total seed FA were significantly associated with SNPs including T/A, G/A, C/A, and T/C at positions 166, 188, 222, and 226, respectively, on gene locus *BnaC*.*TT2*.*a* (Fig 5). All these changes were observed in intron (1). There is a significant association of the four SNPs on introns of *BnaC*.*TT2*.*a* with C18:1 and C18:2 as well as with the long chain FA C22:0 and C22:1, respectively, indicating the underlying role of *BnaC*.*TT2*.*a* in regulating the unsaturated FA composition of *Brassica* seeds. Moreover, significant associations (*P* value = 0.0188, *R*^2^ = 0.09459) of an *Indel* (insertion) at position 738 on Exon 3 of *BnaC*.*TT2*.*a* locus were observed with a*, C18:2, and total FA. These associations indicate that alteration or mutation (SNP) at this site could possibly trigger a functional change in the regulation of seed coat color and composition (C18:2) and total seed oil content in *BnaC*.*TT2*.*a* (Table 6). However, T/G change at position 790 of Exon 3 was significantly correlated with seed color traits b*, L, H°, CIRG, and C18:2. The mutation at position 790 (T/G) caused significant changes in the amino acid (Phe/Ser) that could be significantly associated with seed coat color (Table 6). In contrast, we did not find any significant association between this position of *BnaC*.*TT2*.*a* and total seed oil content.

**Table 6:** DNA Polymorphism (SNPs and/or *Indels*) at *BnaC*.*TT2*.*a* gene locus and their association with seed coat color and FA content and composition among 83 *B*. *napus* genotypes. “#” represents the position need to be checked.

Gene	Position	Polymorphisms	Location	Trait	2010–2011		
					F	*P*	R^2^
*BnaC*.*TT2*.*a*	166	T/A	Intron	C18:1	14.43	2.80E-04	0.1512
				C18:2	4.51	3.68E-02	0.0527
				C22:0	13.38	4.50E-04	0.1417
				C22:1	6.18	1.50E-02	0.0709
				TotalFA	4.72	3.27E-02	0.0551
	188	G/A	Intron	C18:1	25.31	0.00E+00	0.2381
				C18:2	14.98	2.20E-04	0.1561
				C22:0	5.15	2.59E-02	0.0598
				C22:1	14.52	2.70E-04	0.152
				TotalFA	7.54	7.40E-03	0.0852
	222	C/A	Intron	C18:1	25.31	0.00E+00	0.2381
				C18:2	14.98	2.20E-04	0.1561
				C22:0	5.15	2.59E-02	0.0598
				C22:1	14.52	2.70E-04	0.152
				TotalFA	7.54	7.40E-03	0.0852
	226	T/C	Intron	C18:1	25.31	0.00E+00	0.2381
				C18:2	14.98	2.20E-04	0.1561
				C22:0	5.15	2.59E-02	0.0598
				C22:1	14.52	2.70E-04	0.152
				TotalFA	7.54	7.40E-03	0.0852
	738	Indel	Exon	a*	3.93	2.35E-02	0.0895
				C18:2	3.33	4.07E-02	0.0769
				Total_FA	4.18	1.88E-02	0.0946
	790#	T/G	Exon	b*	6.41	2.60E-03	0.138
				L*	6.35	2.80E-03	0.137
				H^o^	5.75	4.60E-03	0.1257
				CIRG	5.21	7.50E-03	0.1153
				C18:2	3.26	4.35E-02	0.0754

## Discussion

Commercial rapeseed varieties differ in seed color from brown to dark brown or black. Breeders have been interested in yellow-seeded genotypes for decades because of their low anti-nutritive elements and higher accumulation of seed FA and protein [1]. *Arabidopsis TT2* prevent FA biosynthesis from developing embryos by directly binding to *FUSCA3* at its regulatory region and mediating the expression profile of several genes involvedin the FA biosynthesis [6]. These downstream genes include *BCCP2*, *CAC2*, *MOD1*, and *KASII*, which regulate the initial steps of FA chain synthesis. Moreover, *FAD2* and *FAD3* are mainly involved in FA desaturation, whereas, *FAE1* catalyzes the chain elongation process of FA. The quantity of PAs in the *Arabidopsis* seed coat negatively correlates with the accumulation of FA in the embryo. *Arabidopsis* and *Brassica* belong to the same family (Cruciferae). Thus, a relationship between the allelic variation of a *BnTT2* locus and the rapeseed phenotypic traits, such as seed coat color, FA content, and composition, is speculated. Our results clearly suggest that *BnaC*.*TT2*.*a* associates with seed color and seed FA accumulation in a collection of 83 rapeseed germplasm.

Rapeseed has an amphidiploid genome that originated from interspecific hybridization between *B*. *rapa* and *B*.*oleracea*. Normally, 2 to 6 homologous copies of a gene are located in different sub-genomes (A or C), and different nucleotide sequences exist among various homologous copies. We aligned the *B*. *napus TT2* sequence with the sequences in the *B*. *napus* genome database, and found its best match in *C08g07960D*, which is allocated to chrC08 from 11760227 bp to 11761328 bp. The match was referred to as *BnaC*.*TT2*.*a* following the standardized gene nomenclature for *B*. *napus* by Østergaard and King (2008) [45]. The gene consists of three exons and two introns (Ref: http://www.genoscope.cns.fr/blat-server/cgi-bin/colza/webBlat).

Direct Sanger sequencing of allopolyploid species such as *B*. *napus* often results in insufficient sequence quality for SNP detection because of existing homologous genes [36, 46]. Fortunately, we used a pair of published primers that was efficient enough to amplify a single PCR fragment and could generate a high quality sequence chromatograph [15]. We successfully cloned a single copy of *BnaC*.*TT2*.*a* to ensure the accuracy of SNP detection. Successful application of this approach has also been reported in previous studies [46–48]. Sequencing data revealed significant polymorphism at the *BnaC*.*TT2*.*a* locus among the 83 *B*. *napus* genotypes, which were categorized into seven haplotype groups based on the accession “DQ778645.1” (Table 4). The SNPs that we detected were both monomorphic and polymorphic. This information enabled us to link the seed phenotypic traits, such as seed coat color and FA, to the genotypic variations evaluated through the DNA sequence alignment of each specimen from the entire germplasm population. We conducted association mapping, for which LD was determined. Phenotypic traits were associated with genotypic variations by applying the GLM + Q model. The blend of phenotypic and genotypic trait associations provided a high number of markers that helped us to accurately investigate the genome-wide diversity and the extent of LD in rapeseed. For instance, previously 845 AFLP markers were used to analyze the extent of LD in 85 winter rapeseed lines. The markers revealed that the highest LD extension in evaluating canola-quality rapeseed was at 2 cM [35]. Moreover, winter rapeseed genotypes were successfully differentiated from other genotypes among 509 inbred lines based on their release dates, levels of erucic acid, and glucosinolates, by analyzing the extent of LD with 89 SSR markers [49]. Furthermore, LD decay within 0.5–1 cM at the genome level was analyzed using 451 SSR markers. The analysis resulted in considerable categorization of a worldwide collection of 192 inbred lines of *Brassica* based on population size, genetic background, and genetic drift [50]. Similarly, associative transcriptomics of 53 *B*. *napus* lines using >60 K SNPs were analyzed by LD. LD analysis identified the transcription factor HAG1 (*At5g61420*), which regulates the biosynthesis of aliphatic glucosinolate in *A*. *thaliana* [51]. Association mapping is conducted based on the LD when regions of the genome are inherited together at a frequency higher than expected based on recombination [52]**.** In our study, LD analysis resulted in the classification of the population into haplotypes, which we further categorized into two groups (G) based on SNP configurations obtained. The groups included haplotypes H0, H1, and H2 as G1 and haplotypes H3, H4, H5, and H6 as G2. G2 genotypes were observed to have lower oil content and inferior oil quality compared with G1 genotypes. The higher C22:1 level and lower C18:2 and C18:1 composition resulted in higher oil quality in G2 genotypes than that of G1. These properties provide the basis for the selection of genotypes with desirable traits for efficient rapeseed breeding.

*R*^2^ is the most relevant LD measurement used to identify the significant association between SNPs or haplotypes and phenotypic trait variations. Typically, *R*^2^ values of 0.1 or 0.2 are often used to describe LD decay. However, we only considered SNPs with frequencies ≥ 0.05 for LD decay analysis to avoid ambiguity. We observed six SNPs and one *Indel*, four of which were located within the first intron and two within the coding sequence (Exon 3) of *BnaC*.*TT2*.*a***.** To that end, 33 significant associations (P < 0.05) were observed for six polymorphic sites (Table 6) on *BnaC*.*TT2*.*a* locus. Out of all those associations, an insertion at position 738 on Exon 3 of *BnaC*.*TT2*.*a* was associated with the color index for redness (a*), C18:2, and total FA. Interestingly, the T/G change at position 790 of Exon 3 was significantly correlated with seed color traits b*, L, H°, CIRG, and FA composition C18:2. This mutation at position 790 resulted in an amino acid (Phe/Ser) change and could significantly be associated with multiple seed traits, particularly the yellowness (b*) of the seed coat (Table 6). The amino acid conversion resulted in the functional change of the protein. This result indicates that *BnaC*.*TT2*.*a* is involved in the regulation of seed oil and seed coat color traits, thereby providing a promising significance for rapeseed breeding. Recently, researchers have focused on applying association mapping to evaluate a broad range of plant species in terms of gene(s) identification that could be responsible for the variation in complex quantitative traits with agricultural and evolutionary perspective. Identification of a gene’s function could be done using transgene technology. We successfully developed molecular markers to improve seed oil quality and regulate seed color using *BnaC*.*TT2*.*a* as a candidate gene in *B*. *napus*.

Regulating the biosynthesis of color-inducing phenolic compounds, such as PA, is widely studied in *A*. *thaliana*. However, the amphidiploid or allotetraploid nature of *B*. *napus* and its extensive gene duplication pose a great challenge in laying out the map of candidate genes responsible for seed pigmentation. Despite these limitations, association mapping shows promising features that could help elucidate the genetic basis of complex traits, which are of qualitative, economic, and ecological importance. This is the first study to investigate the candidate genes behind the *B*. *napus* seed color and oil traits by association mapping. Our results could broaden the scope of research on seed coat color. Our study showed that quantitative genetic approaches, such as association mapping, could improve map-based cloning of key genes for targeted (desirable or undesirable) seed metabolites or compounds in *B*. *napus*.
